# Will the Displacement of Zebra Mussels by Quagga Mussels Increase Water Clarity in Shallow Lakes during Summer? Results from a Mesocosm Experiment

**DOI:** 10.1371/journal.pone.0168494

**Published:** 2016-12-22

**Authors:** Xueying Mei, Xiufeng Zhang, Sinan-Saleh Kassam, Lars G. Rudstam

**Affiliations:** 1 College of Resources and Environment, Anhui Agricultural University, Hefei, China; 2 Cornell Biological Field Station, Department of Natural Resources, Cornell University, Bridgeport, New York, United States of America; 3 Department of Ecology and Institute of Hydrobiology of Jinan University, Guangzhou, China; Stockholm University, SWEDEN

## Abstract

Zebra mussels (*Dreissena polymorpha*) are known to increase water clarity and affect ecosystem processes in invaded lakes. During the last decade, the conspecific quagga mussels (*D*. *rostriformis bugensis*) have displaced zebra mussels in many ecosystems including shallow lakes such as Oneida Lake, New York. In this study, an eight-week mesocosm experiment was conducted to test the hypothesis that the displacement of zebra mussels by quagga mussels leads to further decreases in phytoplankton and increases in water clarity resulting in increases in benthic algae. We found that the presence of zebra mussels alone (ZM), quagga mussels alone (QM), or an equal number of both species (ZQ) reduced total phosphorus (TP) and phytoplankton Chl *a*. Total suspended solids (TSS) was reduced in ZM and ZQ treatments. Light intensity at the sediment surface was higher in all three mussel treatments than in the no-mussel controls but there was no difference among the mussel treatments. There was no increase in benthic algae biomass in the mussel treatments compared with the no-mussel controls. Importantly, there was no significant difference in nutrient (TP, soluble reactive phosphorus and NO_3_^-^) levels, TSS, phytoplankton Chl *a*, benthic algal Chl *a*, or light intensity on the sediment surface between ZM, QM and ZQ treatments. These results confirm the strong effect of both mussel species on water clarity and indicate that the displacement of zebra mussel by an equivalent biomass of quagga mussel is not likely to lead to further increases in water clarity, at least for the limnological conditions, including summer temperature, tested in this experiment.

## Introduction

The spread of invasive *Dreissena* mussels across North America is a matter of concern. The zebra mussel (*Dreissena polymorpha*) and the related quagga mussel (*Dreissena rostriformis bugensis*) are among the most aggressive invaders of freshwater habitats in the northern hemisphere, sometimes reaching densities >10 000 ind. m^-2^ in localized areas [1]. The zebra mussel was first detected in the Great Lakes in 1986 [2] and by 2010, had colonized a total of 729 inland lakes, reservoirs, impoundments, and quarries across the USA and Canada [3,4]. The spread of quagga mussels has been slower. The quagga mussel was first recorded in Lake Erie in 1989 [5] and by 2010 had colonized 43 North American water bodies [4]. However, when quagga mussels arrive they displace previously established zebra mussel populations [4]. The quagga mussel is now the dominant species in some ecosystems, including the Laurentian Great Lakes [6,7] and New York Finger Lakes [8]. While both species continue to spread in both North America and Eurasia [4,9], it is anticipated that the quagga mussel will eventual displace zebra mussels in most of its range [10].

Both species of invasive *Dreissena* are ecosystem engineers that alter the structure and function of the freshwater ecosystems they have colonized [4,11]. The impacts on invaded ecosystems include reduced biomass of phytoplankton, increased water clarity, changed bottom physical structure, and increased benthic algae biomass, which all affect trophic relationships, nutrient cycling, and benthic primary production [4,12,13]. Though the two species appear to have similar overall ecological impact, any differences in performance may have large consequences for invaded ecosystems [14]. For example, the increase in quagga mussels, not zebra mussels, is implicated in the decline of the spring bloom even in large lakes like Lake Michigan [15]. It is likely that even in ecosystems previously invaded by zebra mussels, a dominance shift in favor of quagga mussels may have significant consequences [16] including further increases in water clarity [17]. However, we do not know if this is because quagga mussels can build larger populations as this species can colonize soft substrates, or if the larger effect is due to higher impact of an equivalent biomass of quagga mussels compared to zebra mussels. There are some indications that the quagga mussel filters at higher rates than the zebra mussel [18], especially in the presence of predators [19], although others have observed lower filtering rates of quagga mussels than zebra mussels [20,21]. Without better information on the relative effects of the two species, it is difficult to predict the likely ecological impact of this invasive succession in shallow lake ecosystems [4,10].

The large research effort invested in documenting the ecosystem impacts of invading zebra mussel make this species one of the best studied freshwater invertebrate [22]. Less is known about the quagga mussel and its impacts as an invader [10]. Because most of the waterbodies invaded by quagga mussels have previously been impacted by zebra mussels, it is difficult to separate the effects of the two species using field data. This hinders our ability to predict the likely impact of quagga mussels themselves on specific ecological parameters such as water clarity [4].

Oneida Lake is an ideal ecosystem in which to study the displacement of zebra mussels by quagga mussels. The lake is the largest and best studied inland shallow lake in New York State, with a surface area of 206.7 km^2^, a mean depth of 6.8 m and a maximum depth of 16.8 m [23]. Zebra mussels invaded the lake in 1991, and densities have since ranged from 3600 ind. m^-2^ to 18000 ind. m^-2^, with profound effects on the entire lake ecosystem [10,23,24,25]. Quagga mussels were present in Oneida Lake as early as 2006 [26], and by 2010 the species represented over 90% of the mussel biomass in water deeper than 3 m [21].

In this study, mesocosm experiments were conducted to test the hypothesis that displacement of zebra mussels by quagga mussels will further increase water clarity in these mesocosms, and by extension in Oneida Lake and other shallow lake ecosystems. We further hypothesized that this increase in water clarity would be due to a decrease in phytoplankton Chl *a* and perhaps TSS, and lead to increases in benthic algae. The experiment was conducted for two months in the summer and therefore apply to summer conditions.

## Materials and Methods

### Experimental setup

“N/A.—No specific permissions were required for these locations/activities as our experiments did not involve vertebrates or endangered and protected species. The two mussels species are invasive species in the US and were euthanized after the experiments were completed as required by NY State law.”

In this study, mesocosm experiments were conducted in 200-L containers with water from Oneida Lake and stocked with zebra mussels (ZM), quagga mussels (QM), or both species at equal densities (ZQ). All three mussel treatments had similar total mussel density and biomass. Tanks without mussels served as controls. Response variables included phytoplankton, benthic algae, total suspended solids (TSS), nutrient concentrations (total phosphorus, soluble reactive phosphorus, and nitrate) and light intensity on the sediment surface.

Sixteen mesocosms were established in plastic tanks (upper diameter = 57 cm, bottom diameter = 56 cm, and height = 79 cm) using water and sediments from Oneida Lake. Sediments were collected from a boat (from June 5 to 19, 2015) using a Petite Ponar sampler, and passed through a 0.5 mm mesh sieve to remove mussels, coarse debris and clumps before being added to the mesocosms in a layer ~5 cm thick. The tanks were then each filled with 160 L Oneida Lake water filtered through a 64 μm mesh plankton net. The filtered lake water contained (mean ± 1SD) 19.9 ± 2.6 μg L^-1^ total phosphorus (TP), 7.0 ± 0.2 μg L^-1^ soluble reactive phosphorus (SRP), 178.8 ± 19.4 μg L^-1^ NO_3_^-^, 2.6 ± 1.3 μg L^-1^ Chlorophyll *a* (Chl *a*) and 13.3 ± 0.6 μg L^-1^ total suspended solids (TSS). The mesocosms were set in natural sunlight at the Cornell Biological Field Station close to Oneida Lake and allowed to acclimatize for one week before the experiments began. The tanks were not mixed during the experiment. At the end of the acclimatization period, the concentrations in the mesocosms were: 17.8 ± 2.7 μg L^-1^ TP, 2.3 ± 0.4 μg L^-1^ SRP, 45.3 ± 3.3 μg L^-1^ NO_3_^-^, 2.9 ± 1.1 μg·L^-1^ Chl *a* and 7.4 ± 2.6 mg L^-1^ TSS. There are several possible reasons for the decline in SRP and NO_3_^-^ during the acclimatization period, including uptake by phytoplankton and benthic algae on the sediment and tank walls as well as adsorption to organic and inorganic particles that settled to the sediment. But whatever the cause, we consider the concentration at the end of the acclimatization period to be the initial conditions of our experiment.

After acclimatization, a petri dish (diameter 5.3 cm) filled entirely with sieved sediment was inserted into the sediment layer of each mesocosm, such that the sediment surfaces inside and outside the petri dish were level, allowing benthic algae to colonize freely. Zebra mussels (wet weight: 0.37 ± 0.18 g ind^-1^, length 14.3 ± 2.1 mm) and quagga mussels (wet weight: 0.39 ± 0.16 g ind^-1^, length 14.9 ± 1.9 mm) were collected from Oneida Lake. Twenty live zebra mussels were added to each of four mesocosms designated as zebra mussel (ZM) treatments. Likewise twenty live quagga mussels were used to populate the quagga mussel (QM) mesocosms. Another four mesocosms each received ten mussels of each species (ZQ). Each mussel used in the experiment was marked with an individual number attached with super glue. The mussels were placed on the sediment surface and allowed to move around freely during the experiment. Zebra mussels tended to move to the wall and attach at the wall-sediment interface whereas quagga mussels moved around but stayed on the sediment surface. The remaining four mesocosms were maintained as mussel-free controls. The tanks were visually checked every other day during the experimental period and no obvious signs of morbidity or mortality of mussels were observed until the last week of the experiment. At that time, between one and six individuals from each mesocosm were dead or missing. All sixteen tanks received weekly supplements of nitrogen (N) and phosphorus (P) to mimic the external nutrient loading of Oneida Lake [27]. P was added as a solution of NaH_2_PO_4_ at a rate equivalent to 3.2 mg P mesocosm^-1^ wk^-1^, and N was added as KNO_3_ at a rate of 48.5 mg N mesocosm^-1^ wk^-1^. Tanks were topped up with filtered lake water as necessary to maintain a constant water level in the mesocosms and a total of about 15 L filtered lake water was added to each mesocosm representing a minimal additional input of TP to the tanks (0.3 mg from the added water compared to 25.6 mg from the nutrient additions). The experiment ran from June 30, 2015 to August 24, 2015.

### Sampling and analysis

Light intensity at the sediment surface of each mesocosm was measured every two weeks prior to the sampling of benthic algae, using a portable lux meter (MW700). Water temperature was measured using a Hydrolab Datasonde II. In addition, key factors affecting water clarity were measured, including TP, SRP, TSS, phytoplankton biomass and benthic algal biomass. Although P was the main nutrient of interest in this study, we also measured NO_3_^-^ as one of the key inorganic nitrogen sources.

Water samples (1L) were collected every two weeks during the experiment from 20–30 cm under the water surface in the middle of each mesocosm and used for analysis of TP, SRP and NO_3_^-^ levels, phytoplankton biomass and TSS. We did not mix the mesocosms before sampling. Chlorophyll *a* (Chl *a*) as a proxy for phytoplankton biomass was measured after acetone extraction at room temperature using a Turner Trilogy ® fluorometer calibrated using a standard with a known Chl *a* concentration. TP was measured according to Menzel and Corwin [28]. SRP was measured using the same method as TP, but after filtering with GF/C filters (0.45 μm) and without persulfate digestion. NO_3_^-^ was analysed according to APHA [29]. TSS was determined from the dry mass of material retained on GF/C grade filters from 500 ml of mesocosm water. Filters were dried at 105°C for 24h to calculate TSS.

The petri dishes, complete with their sieved sediment and two weeks’ accumulation of benthic algae were removed from each mesocosm after sampling of water, and replaced immediately by fresh sediment-filled dishes. Benthic algae growing on the sediment surface in each dish were collected by scraping with a razor blade [30]. As with phytoplankton, Chl *a* levels were measured as phytoplankton and used as an indicator of benthic algal biomass.

Wet weight and length was measured for each individual mussel at the start and at the end of the experiment. The length was measured by a caliper to the nearest 0.1 mm and the wet weight biomass was determined with a balance to the nearest 0.001 g. Daily growth rate was calculated as the difference between the measurements at the start and at the end of the experiment divided by the duration of the experiment.

### Statistical analyses

Repeated measures analyses of variance (RM-ANOVA) was used to evaluate the effects of different treatments on nutrient levels, phytoplankton biomass, TSS, light intensity and benthic algal biomass, with time as the repeated factor. An LSD test was used to detect differences between treatments when the overall model was significant. One-way ANOVA was used to detect differences among treatments on each sampling occasion. If the difference was significant, an LSD test was performed to detect which treatments differed. All statistical analyses were performed with SPSS 16.0 software (SPSS, USA). All results are presented as mean ± 1SD.

## Results

### Survival and growth rate

Survival was relatively high throughout the experiments and most death occurred at week 8. Even so, 76% of zebra mussels in ZM treatment and 86% of quagga mussels in QM treatment survived the 8 week experiment. In the ZQ treatment, 78% of zebra mussels and 88% of quagga mussels survived for 8 weeks (Table 1).

**Table 1:** Average wet weight biomass (g), individual length (mm), growth rate (mm d^-1^) and survival of mussels used in the mesocosm experiments (mean±1SD).

Treatments	Biomass (g)		Growth rate (mg d^-1^)	Length (mm)		Growth rate (mm d^-1^)	Survival
	at start	at end		at start	at end		
ZM	0.36±0.18	0.57±0.19	3.2±1.8	14.1±2.1	16.0±1.8	0.027±0.019	76%
QM	0.38±0.15	0.45±0.16	1.0±0.6	14.9±1.8	15.4±1.8	0.007±0.009	86%
ZQ-zebra	0.39±0.20	0.54±0.18	2.8±1.6	14.5±2.2	15.±1.8	0.022±0.014	78%
ZQ-quagga	0.40±0.19	0.49±0.19	1.4±0.8	14.9±2.1	15.4±2.0	0.009±0.010	88%

Growth rates were calculated as the average growth of individual mussels alive at the end of the experiment.

Zebra mussels grew faster than quagga mussels in both biomass and length (one-way ANOVA, treatment effect, *p*< 0.05, Table 1). The growth rate of zebra mussels was 3.16 ± 1.75 mg d^-1^ or 0.027 ± 0.019 mm d^-1^ in the ZM treatment and 2.76 ± 1.58 mg d^-1^or 0.022 ± 0.014 mm d^-1^ in the ZQ treatment. In contrast, the growth rate of quagga mussels was 0.99 ± 0.56 mg d^-1^or 0.007 ± 0.009 mm d^-1^ in the QM treatment and 1.40 ± 0.81 mg d^-1^ or 0.009 ± 0.010 mm d^-1^ in the ZQ treatment.

### Light and temperature

**Fig 1:**
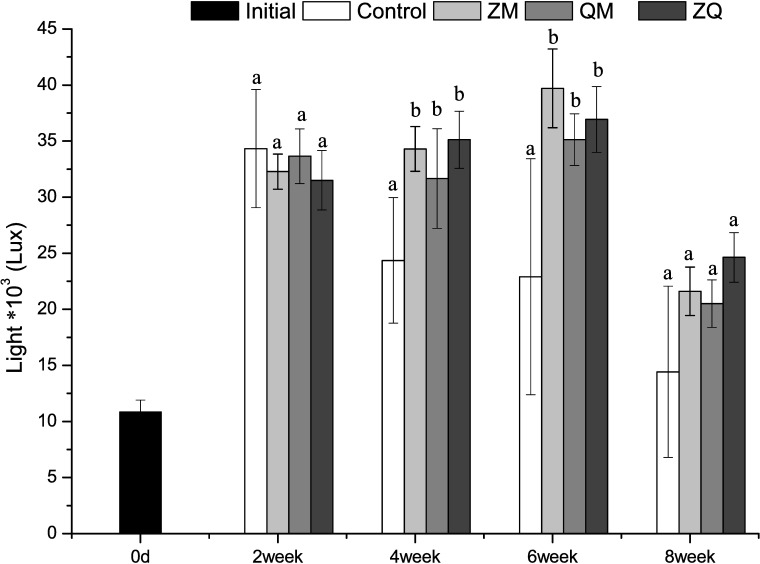
Mean light intensity at the sediment surface in different treatments. Error bars indicate 1 SD. Letters a, b indicate significant (*p*< 0.05) differences. Treatments that are not significantly different share a common letter.

Light intensity at the sediment surface was higher in all the mussel treatments than in the controls (RM-ANOVAs, treatment effect, F_3, 12_ = 4.94, *p* = 0.018; Fig 1). However, we did not detect any significant difference between the three mussel treatments (*p*>0.05). Analyses of the effects of mussels at each sampling event revealed that the light intensity was higher in the mussel treatments than in the control from week 4 to week 6 (one-way ANOVA, treatment effect, *p*< 0.05).

Water temperature in the mesocosm was around 19°C at the start of the experiment, then increased to between 28 and 31°C in weeks 2 to 6. By week 8, temperatures declined to 23°C (Table 2). There was no significant difference in water temperature among treatment groups on any sampling occasion (one-way ANOVA, treatment effect, p> 0.05).

**Table 2:** Water temperature (°C, mean ± 1SD) in the different treatments during the experiment.

Treatments	0 week	2 week	4 week	6 week	8 week
Control	18.6 ± 0.1	31.2 ± 0.2	30.2 ± 0.4	27.8 ± 1.0	23.2 ± 0.6
ZM	18.8 ± 0.2	31.3 ± 0.3	30.3 ± 0.2	27.8 ± 0.5	23.0 ± 0.2
QM	18.8 ± 0.0	31.3 ± 0.5	30.3 ± 0.9	28.2 ± 1.4	23.3 ± 0.6
ZQ	19.0 ± 0.2	31.3 ± 0.2	30.3 ± 0.7	27.9 ± 1.1	23.1 ± 0.4

### Nutrients

**Fig 2:**
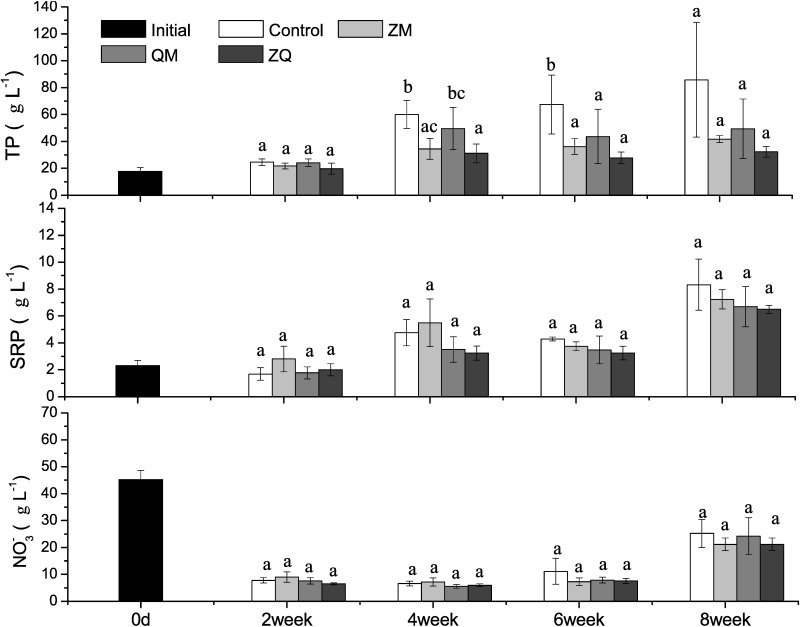
Mean total phosphorus (TP), soluble reactive phosphorus (SRP) and nitrate (NO_3_^-^) values in different treatments over time. Error bars indicate 1 SD. Letters a, b indicate significant (*p*< 0.05) differences in TP. Treatments that are not significantly different share a common letter.

TP values differed among treatment groups (RM-ANOVAs, treatment effect, F_3,12_ = 5.97, *p* = 0.010), being lower in ZM (*p* = 0.007), QM (*p* = 0.046) and ZQ (*p* = 0.002) treatments than in the controls with the most pronounced effects in the ZQ treatments. TP levels also varied significantly with time (RM-ANOVAs, time effect, F_3,12_ = 18.1, *p*<0.0001). TP levels in the QM treatment at week 6 and in both the ZM and ZQ treatments by week 4 and 6 were significantly lower than in the controls (one-way ANOVA, treatment effect, *p*<0.05, Fig 2).

Overall, concentrations of SRP (RM-ANOVAs, time effect, F_3,12_ = 116, *p*<0.001) and NO_3_^-^ (RM-ANOVAs, time effect, F_3,12_ = 140, *p*<0.001) increased over the course of the experiment. However, there was no significant difference in SRP (RM-ANOVAs, treatment effect, F_3,12_ = 3.07, *p* = 0.069) or NO_3_^-^ (RM-ANOVAs, treatment effect, F_3,12_ = 1.51, *p* = 0.262) concentrations among treatments. On no sampling occasion did values of either SRP or NO_3_^-^ differ significantly among mussel treatments and controls (one-way ANOVA, treatment effect, *p*>0.05). At the end of the experiments TP was around 40 μg L^-1^ (higher in controls), SRP around 7 μg L^-1^ and NO_3_ around 20 μg L^-1^, which are levels similar to September concentrations in Oneida Lake in 2015 (TP 21–35 μg L^-1^, SRP 1–19 μg L^-1^, NO_x_ 4–24 μg L^-1^ [23].

### Phytoplankton and TSS

**Fig 3:**
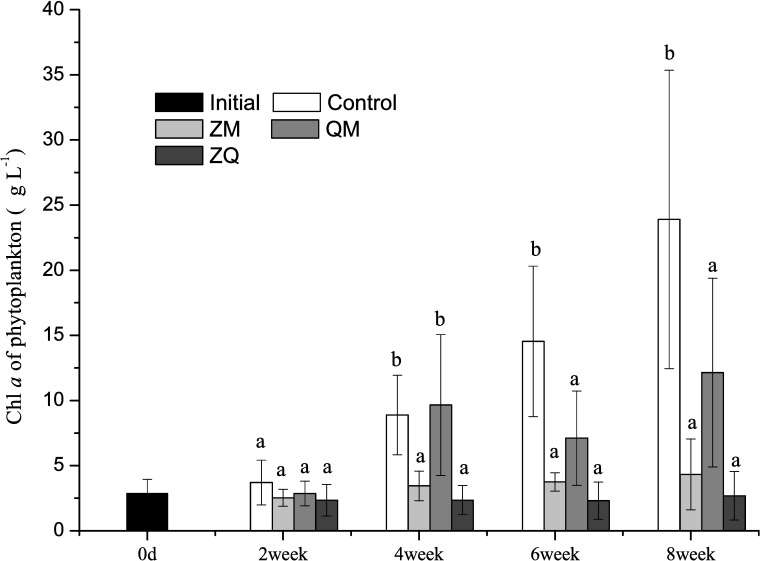
Mean Chl *a* levels indicating phytoplankton abundance in different treatments over time. Error bars indicate 1 SD. Letters a, b, c indicate significant (*p*< 0.05) differences in Chl *a*. Treatments that are not significantly different share a common letter.

Phytoplankton Chl *a* levels were consistently lower in all mussel treatments than in the controls (RM-ANOVAs, treatment effect, F_3, 12_ = 9.79, *p* = 0.002; Fig 3), indicating that both zebra and quagga mussels depress phytoplankton abundance. However, no significant difference of the recorded Chl *a* values was found between ZM, QM and ZQ treatments (*p*>0.05).

Phytoplankton Chl *a* levels also changed significantly over time (RM-ANOVAs, time effect, F_3, 12_ = 14.1, *p*<0.0001) and disparities became apparent from week 4 onwards. By week 4, Chl *a* was lower in the ZM and ZQ treatments than in the controls (one-way ANOVA, treatment effect, *p*<0.05) and this difference increased until the end of the experiment. At weeks 6 and 8, Chl *a* values in the QM treatments also were significantly below those observed in the controls (one-way ANOVA, treatment effect, *p*< 0.05).

**Fig 4:**
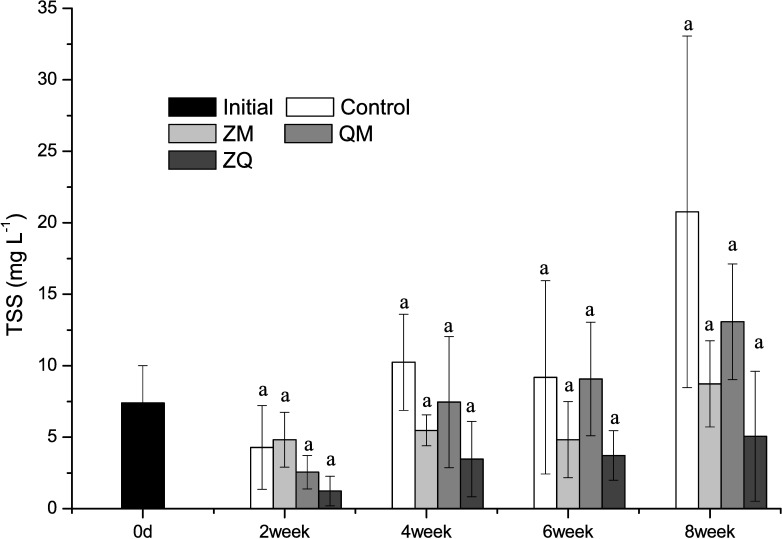
Mean total suspended solids (TSS) in different treatments over time. Error bars indicate 1 SD. Treatments that are not significantly different share a common letter.

In general, TSS mirrored those in Chl *a* and increased significantly over time (RM-ANOVAs, time effect, F_3, 12_ = 14.4, *p*<0.001). Differences in TSS were also observed between treatment groups (RM-ANOVAs, treatment effect, F_3, 12_ = 5.11, *p* = 0.017; Fig 4). TSS was lower in the ZM (*p* = 0.027) and ZQ (*p* = 0.003) treatments than in the controls, but the differences between QM treatments and controls (*p*>0.05) were not statistically significant. In addition, the differences between mussel treatment groups and controls on any given sampling occasion were not significant (one-way ANOVA, treatment effect, *p*>0.05).

### Benthic algae

**Fig 5:**
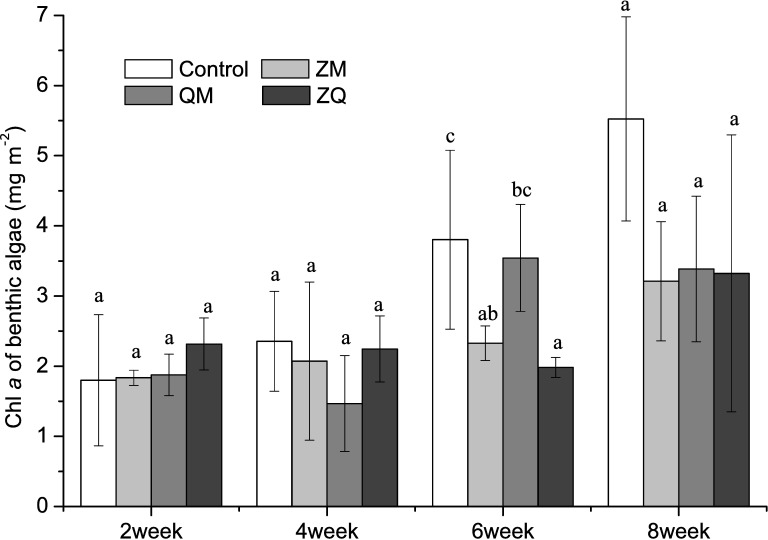
Mean Chl *a* from benthic algae in different treatments over time. Error bars indicate 1 SD. Letters a, b indicate significant (*p*< 0.05) differences in Chl *a*. Treatments that are not significantly different share a common letter.

Unlike phytoplankton in the overlying water, benthic algal Chl *a* concentration did not differ significantly among the mussel treatments and the controls (RM-ANOVAs, treatment effect, *p*>0.05; Fig 5). Benthic algal Chl *a* levels varied significantly with time (RM-ANOVAs, time effect, F_3, 12_ = 21.8, *p*<0.001). Levels appeared to rise more rapidly in the control tanks, but the differences observed between treatments throughout the experiment were statistically significant only at week 6 when the ZM and the ZQ treatments both exhibited lower benthic Chl *a* than the controls (one-way ANOVA, treatment effect, *p*<0.05).

## Discussion

We found that concentrations of TP and phytoplankton Chl *a* levels in all mussel treatments were reduced relative to the controls. TSS was also reduced in the ZM and ZQ treatments, but not in the QM treatment. This was contrary to our initial hypothesis. Our results suggest that the displacement of zebra mussel by the same biomass of quagga mussels is unlikely to lead to additional increases in water clarity, at least for the limnological conditions, including the summer temperature, tested in our experiment. Interestingly, the observed increases in light intensity at the sediment surface associated with the presence of mussels did not result in any significant increase in benthic algal Chl *a*.

The impact of zebra and quagga mussels on water clarity is associated with their role as filter feeders. By filtering out particulate matter, these mussels can reduce both phytoplankton biomass and particle-bound nutrients in the water column [11,31,32,33]. This was also the case in our experiment as both TP and Chl *a* were lower in the treatments than in the controls. These lower TP levels in the water in mussel treatments are likely due to deposition of material-bound P in feces and pseudofeces on the sediment and/or to P stored in the mussels due to the increased mussel biomass at the end of the experiment (Table 3). In these experiments, 25.6 mg P was added during the 8 weeks to each mesocosm to mimic nutrient loading in Oneida Lake. Only 9–20% of the added P remained in the water in the mussel treatments, compared to 42% in the controls. Of the added P, 3–10% could be accounted for by mussel growth (assuming a P content of 9.2 ± 1.5 mg gdwt^-1^ in the soft tissue of dreissenid [34]). Therefore, considerably more of the added P likely ended up in the sediment and possibly in periphyton growing on the wall of the mesocosms when mussels were present than in the controls (Table 3). By contrast we did not detect a significant effect of mussels on SRP and NO_3_^-^ in the water column in this experiment. Thus we could not detect an increase in SRP as a result of mussel excretion, similar to the results of Bruesewitz [35]. Our conclusions about nitrogen excretion by mussels are more uncertain because we did not measure ammonia in the mesocosm. More research is needed on the differences in nitrogen excretion between the two mussel species.

**Table 3:** Increase of P (mg) in the later and mussel tissue in the different experimental treatments during the experiment.

Treatments	Water	P in added mussel tissue	Sediment
Control	10.9 (42%)	0 (0%)	14.7 (58%)
ZM	3.8 (15%)	1.7 (7%)	20.1 (78%)
QM	5.1 (20%)	0.7 (3%)	19.8 (78%)
ZQ	2.3 (9%)	2.4 (10%)	20.8 (81%)

The amount of P in the sediment (including periphyton growing on the wall of mesocosms) was obtained through the difference between the total amount of added P (25.6 mg) and the increase in the water and tissue. Calculations of the amount of P in new mussel tissue is based on measured increase in wet weight, 9.2 mg P /g dry weight [34], and a wet to dry weight conversion from Nalepa et al. [50]. The percentage of added P in the different pools are given in parenthesis.

We expected that the higher light levels on the sediment surface observed in our experiment would have benefitted benthic algae [36,37,38]. However, there was no significant increase in benthic algal Chl *a* in the mussel treatments compared to the controls. Perhaps the light intensity at the sediment surface in our experiments was not a limiting factor for benthic algae as the mesocosms were only slightly over 0.5 m deep. Indeed, light levels at the sediment surface at shallow depth might even be photo-inhibitory at the higher water clarity associated with mussels [39,40]. In deeper lakes, light has to be an important factor controlling benthic primary production rates and the distribution of benthic algae [41]. Increased water clarity and light penetration resulting from the presence of the mussels is likely to allow benthic algae to grow deeper and cover larger portions of invaded lake bed [11,13,42]. Previous research in Oneida Lake has demonstrated that the average depth receiving 1% surface light increased from 6.7 m to 7.8 m, representing a 23% areal expansion [25]. It is thus reasonable to expect that larger portions of the lake bottom will be covered by benthic algae than in the pre-mussel years [36,40], even though we did not detect increased benthic algal biomass in our experiments.

Our results suggest that filtering rates of zebra and quagga mussels are comparable since the effect on water clarity and phytoplankton was similar. Experimental comparisons of filtering rates by the two mussels have produced conflicting results. Ackerman [43] did not find a significant difference in clearance rate between *D*. *polymorpha* and *D*. *r*. *bugensis*. Diggins [18] reported that clearance rates in quagga mussels are up to 37% higher than those achieved by zebra mussels. However, Baldwin et al. [20] and Hetherington [21] found the filtration rates of quagga mussels to be lower than those of zebra mussels. Clearance rate can be influenced by many factors, including individual size, temperature and the type and concentration of particles being filtered [44]. The inconsistencies among published clearance rates may be because of different effects of factors modifying clearance rates between the two species. For example, Naddafi and Rudstam [19] found that quagga mussels had higher filtering rates than zebra mussels in the presence of predator kairomones but not when predator kairomones were absent. In the present experiments, we used zebra and quagga mussels of similar size and in similar experimental conditions without the presence of mussel predators. Thus any significant differences in water clarity between treatments would be attributable to effects of species alone. However since no such differences were recorded in our study, we conclude that the displacement of zebra mussel by an equivalent biomass of quagga mussels will not further increase water clarity, at least at the summer temperature tested in this experiment. Zebra mussels have higher upper thermal tolerance than quagga mussel [45] and we did observe slower growth rates of quagga than of zebra mussels (Table 1). It is possible that quagga mussels would have a larger impact than zebra mussels at colder temperatures (spring, fall, winter) as quagga mussels grow and reproduce better in colder temperatures than zebra mussels [5,10].

The presence of mussels may impact water clarity in ways not related to filter feeding. For example, *Dreissena* spp. are known to move while foraging and in response to various environmental factors [46]. They plow through subsurface sediment layers [47], thereby dislodging particulate matter and nutrients from the sediment into the overlying water [32], potentially increasing TSS and stimulating pelagic algal production, both of which may result in increased water turbidity. Peyer et al. [48] observed zebra mussel moving 20 cm within 20 h, while quagga mussel moved 29 cm in the same time. Quagga mussels are also more often found on soft sediment than zebra mussels [44]. Quagga mussels may therefore have a stronger effect on particle and nutrient transport from the sediment than zebra mussels. This may explain the failure of the quagga-only treatment in the present study to reduce TSS, while in the zebra-only treatment TSS was significantly reduced.

The biomass of mussels (29.78 ± 0.47 g m^-2^ for ZM, 31.29 ± 0.71 g m^-2^ for QM and 32.01 ± 0.45 g m^-2^ for the ZQ treatments) used in our mesocosm experiments was lower than that typically occurring in sediments in Oneida Lake (~ 161 ± 94 g m^-2^ on sand and ~166 ± 67 g m^-2^ on silt) [10]. Thus, effects in the lake may be stronger than observed in our experiments. On the other hand, our mesocosm did not contain mussel predators which are known to negatively affect filtration rates of at least the zebra mussels [19, 49], and the mussels were not experiencing physical stress from wave action or other organisms; both factors that may have enhanced the mussel effect in our experiment compared to the lake.

The two species of mussel investigated in this study have now invaded thousands of lakes and rivers throughout Eurasia and North America [11], and the range of both species continues to expand. As quagga mussels are displacing zebra mussels, the potential consequences for water clarity or any other ecological parameter are of considerable interest. Our results indicate that this displacement will not further change the water clarity and associated ecological processes in shallow lakes during the summer unless quagga mussels build up larger lake-wide populations than zebra mussels. Of course, other changes associated with the increase in quagga mussels could also be important, such as effects on habitat complexity and the community structure of zooplankton and phytoplankton.
